# Compliant Micron-Sized Patterned InGaN Pseudo-Substrates Utilizing Porous GaN

**DOI:** 10.3390/ma13010213

**Published:** 2020-01-04

**Authors:** Shubhra S. Pasayat, Chirag Gupta, Yifan Wang, Steven P. DenBaars, Shuji Nakamura, Stacia Keller, Umesh K. Mishra

**Affiliations:** 1 Department of Electrical and Computer Engineering, University of California Santa Barbara, Santa Barbara, CA 93106, USA; chiragg@ece.ucsb.edu (C.G.); spdenbaars@ucsb.edu (S.P.D.); snakamura@ucsb.edu (S.N.); stacia@ece.ucsb.edu (S.K.); mishra@ece.ucsb.edu (U.K.M.); 2 Department of Physics, University of California, Santa Barbara, CA 93106, USA; yifanwang@ucsb.edu; 3 Materials Department, University of California, Santa Barbara, CA 93106, USA

**Keywords:** indium gallium nitride, gallium nitride, porous GaN, relaxed InGaN pseudo- substrate, compliant pseudo-substrate, composition pulling effect, MOCVD

## Abstract

The compliant behavior of densely packed 10 × 10 µm^2^ square patterned InGaN layers on top of porous GaN is demonstrated. The elastic relaxation of the InGaN layers is enabled by the low stiffness of the porous GaN under layer. High resolution X-ray diffraction measurements show that upon InGaN re-growths on these InGaN-on-porous GaN pseudo-substrates, not only was the regrown layer partially relaxed, but the degree of relaxation of the InGaN pseudo-substrate layer on top of the porous GaN also showed an increase in the *a*-lattice constant. Furthermore, methods to improve the surface morphology of the InGaN layers grown by metal-organic chemical vapor deposition (MOCVD) were explored in order to fabricate InGaN pseudo-substrates for future optoelectronic and electronic devices. The largest *a*-lattice constant demonstrated in this study using this improved method was 3.209 Å, corresponding to a fully relaxed InGaN film with an indium composition of 0.056.

## 1. Introduction

The (In,Ga)N alloy system is attractive for various optoelectronic and electronic applications, owing to its wide tunable bandgap spanning from 0.7 to 3.4 eV. While high brightness blue and white light emitting diodes (LEDs) are commercially available, the fabrication of highly efficient (Ga, In) N based green, yellow, or red light emitting diodes (LEDs) still remains challenging [1]. Achieving high indium incorporation into InGaN alloys while maintaining high film quality remains difficult in particular due to the large lattice mismatch of 10% between GaN and InN [2,3]. The misfit strain also leads to reduced indium incorporation through the so-called compositional pulling effect [4]. A suppression of the indium incorporation into compressively strained InGaN films compared to relaxed, strain-free InGaN was found in both experimental as well as thermodynamic studies [5,6,7,8,9,10]. Due to the reduced lattice mismatch between a relaxed InGaN buffer and the quantum wells (QWs), a higher indium incorporation efficiency can be achieved. Typically, the indium composition in the quantum wells (QWs) must stay below 20–25% to maintain good film quality [6]. 

The availability of a relaxed InGaN buffer layer with a lattice parameter closer to that of the QWs is hence highly desirable to obtain high efficiency long wavelength (>500 nm) III-N LEDs or lasers. Attempts have been made to fabricate as-grown relaxed InGaN buffers on substrates such as ZnO [11,12,13] and ScAlMgO_4_ [14,15]; however, the very low growth temperatures required for deposition on ZnO and the high n-type conductivity of InGaN grown on ScAlMgO_4_ substrates have made metal-organic chemical vapor deposition (MOCVD) growth efforts on these substrates very challenging. Alternately, partially relaxed engineered InGaN substrates have been explored [6] and relaxed InGaN buffer layers have been fabricated by molecular-beam epitaxy (MBE) and used as pseudo substrates for MOCVD growth [9,16,17]. Attempts have also been undertaken to fabricate InGaN pseudo-substrates via coalescence of relaxed nano-feature arrays [18]. Additionally, previous theoretical studies have established the advantages of ternary InGaN substrates with enlarged in-plane lattice parameters compared to GaN for the growth of high In-content InGaN QWs [19,20,21,22,23]. 

Prior to this work, 80 to 200-nm wide InGaN fins were shown to uni-axially relax perpendicular to the fin direction [17,24,25]. Recently, we demonstrated uni-axial and bi-axial InGaN relaxation in the micron-sized regime [10] using porous GaN under layers. Using these relaxed InGaN-on-porous-GaN wafers as pseudo-substrates for the growth of InGaN/GaN multi-quantum wells (MQWs) we observed enhanced indium incorporation into the InGaN QWs grown on the pseudo-substrates compared to the same structure grown on GaN base layers. 

In this work, we report on the compliant nature of InGaN layers on top of porous GaN. Upon performing bulk In_y_Ga_1−y_N regrowths on relaxed In_x_Ga_1−x_N-on-porous-GaN pseudo-substrates (where y ≥ x), and measuring the lattice constant of each film, an increase in the lattice constant of the In_x_Ga_1−x_N layer was observed. Furthermore, the pseudo-substrate design was optimized and an InGaN-pseudo-substrate with a lattice constant corresponding to fully relaxed InGaN with an indium composition of 0.0565 and good surface morphology was obtained, which is essential for future electronic and opto-electronic devices.

## 2. Materials and Methods

**Figure 1:**
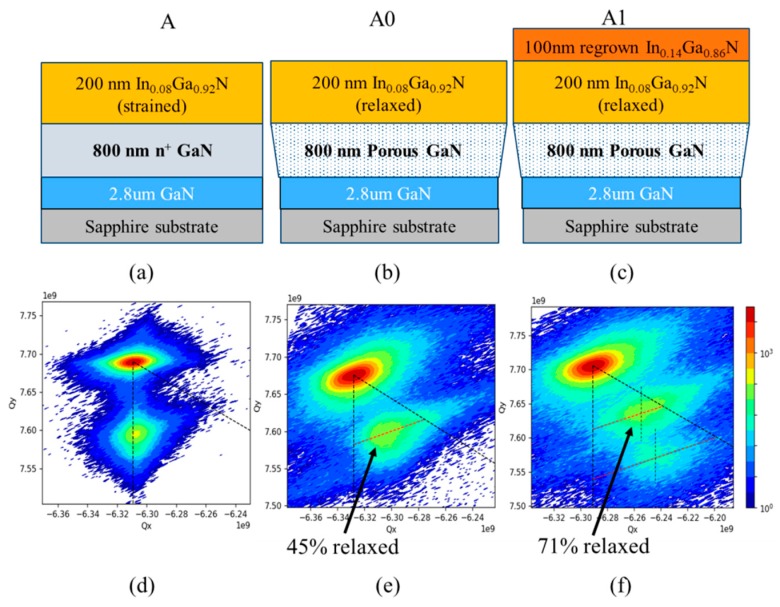
Schematic structure of (**a**) the as-grown sample, (**b**) post porosification, and (**c**) upon regrowth of 100 nm of InGaN, with corresponding RSMs measured using the GaN ($\bar{11}2$4) reflection to evaluate the InGaN relaxation across the tiles shown in (**d**–**f**). The black vertical dashed line through the GaN peak corresponds to the fully strained InGaN lattice constant, and the slanted line through the GaN peak corresponds to fully relaxed InGaN lattice constant in (**d**–**f**). The red dashed lines through the In_x_Ga_1−x_N and In_y_Ga_1−y_N peaks in (**e**) and (**f**), correspond to lines of constant indium mole fraction x or y, where the degree of relaxation varies from 0 to 100% from left to right. The In_x_Ga_1−x_N peak in (**e**) shifted to the right along the red dashed line in (**f**) as a result of regrowth of In_y_Ga_1−y_N layer. The vertical dashed lines through the In_x_Ga_1−x_N and In_y_Ga_1−y_N peaks in (**f**) show a slight offset corresponding to about 20% lattice mismatch between the two layers.

All epitaxial layers in this study were grown by metal-organic chemical vapor deposition using the precursors trimethylgallium (TMGa), triethylgallium (TEG, for InGaN growths), trimethylindium (TMI), ammonia, and disilane on c-plane sapphire substrates. The epitaxial layer structure of the samples for porosification consisted of a 2.8 µm-thick unintentionally doped (u.i.d.) GaN layer followed by 800-nm-thick Si-doped GaN with a doping of 5 × 10^18^ cm^−3^, and a 80 or 200-nm-thick compressively strained In_x_Ga_1−x_N (0.05 ≤ x ≤ 0.125) top layer (Figure 1a). 

The samples were first patterned and dry etched using a 100 W BCl_3_/Cl_2_ etch chemistry, resulting in 10 µm × 10 µm wide tiles. The spacing between each tile was 2 µm, making the fill factor for tiles 10 µm × 10 µm over 12 µm × 12 µm, or 69%. The total etch depth was 580 or 700 nm, corresponding to In_x_Ga_1−x_N layer thicknesses of 80 or 200 nm, respectively. Afterward a doping selective electrochemical (EC) etch was used for the porosification of the exposed regions of the 800 nm thick GaN: Si layers [10]. The EC etch was performed with a metal contact to the 800-nm-thick n+ GaN: Si layer on the sample as anode and a Pt wire which acted as the cathode. The redox reaction resulting in etching progressed as a result of the current flowing through this GaN: Si layer etching the region exposed to the 0.3 M Oxalic acid electrolyte resulting in the formation of tiles comprised of In_x_Ga_1−x_N on top of porous GaN as shown in Figure 1b [26]. Following this process, the samples were used as pseudo-substrates for subsequent regrowth of 100 or 200 nm thick In_y_Ga_1−y_N with TMI and TEG flows of 11.3 and 6.5 μmol/min at 869 °C. A co-loaded GaN-on-sapphire template served as the reference sample for each experiment.

The surface morphology of the samples was assessed using an Asylum MFP3D atomic force microscope (AFM). The V-defect density on the sample surface was determined by counting the V defects over an area of 5 µm × 5 µm from multiple AFM scans and averaged to obtain the V defect density for each sample. The cross-sectional images were taken using a Field Electron and Ion (FEI) Helios Dualbeam Nanolab 600 Focused Ion Beam (FIB) tool operated at 5 kV. High-resolution X-ray diffraction (XRD) (ω–2θ)–ω reciprocal space maps (RSMs) were recorded around the GaN ($\bar{11}2$4) reflection to evaluate the InGaN relaxation across the tiles. In addition, ω–2θ scans around the GaN (0002) reflection were measured, all using an X’PertPro Panalytical Pixcel 3D diffractometer. The lattice constants of the In_x_Ga_1−x_N layer before and after regrowth and the In_y_Ga_1−y_N after regrowth, were determined using the X-pert Epitaxy software using ‘a’ lattice constants of 3.1893 Å and 3.538 Å for GaN and InN, respectively. The lattice constant ‘a_new_’ of an In_z_Ga_1−z_N layer, which was relaxed, R%, was calculated following Vegard’s law:

a_new_ = 3.1893 × {1 − (z × R/100)} + 3.538 × z × R/100. (1)

## 3. Results and Discussion

### 3.1. Experiment 1: In_x_Ga_1−x_N Layer Thickness 200 nm (Sample Series A)

In the first experiment, we investigated the relaxation of a 200 nm-thick In_x_Ga_1−x_N layer with x = 0.08, which was initially grown strained to the GaN base layers (sample A, Figure 1a), after porosification of the GaN:Si underlayer (sample A0, Figure 1b), and after a subsequent regrowth of an additional 100 –nm-thick–In_y_Ga_1−y_N layer with y = 0.145 (sample A1, Figure 1c). Post EC etch at a bias of ~30 V, the In_x_Ga_1−x_N layer with x = 0.08 was ~45% relaxed (Figure 1e) or R = 45, resulting in a lattice constant of 3.202 Å. Upon subsequent regrowth of 100 nm In_y_Ga_1−y_N under the conditions described above, an indium mole fraction of y = 0.145 was measured for the In_y_Ga_1−y_N regrown on the tile sample, compared to only y = 0.12 for the co-loaded GaN-on-sapphire reference sample. 

The enhanced indium uptake can be explained by the composition pulling effect, as the lower misfit strain on the tiles led to a higher indium incorporation in the regrown layer [9]. Interestingly, regrowth of the In_y_Ga_1−y_N layer with a higher indium content (y = 0.145) than that of the In_x_Ga_1−x_N layer underneath (x = 0.08) led to an increase in the degree of relaxation of the In_x_Ga_1−x_N layer from 45% to ~71% as shown in Figure 1f, corresponding to an increase of its ‘a’ lattice constant from 3.202 to 3.209 Å. This result indicates that the use of porous GaN as a mechanically flexible layer allows the In_x_Ga_1−x_N layer on the top to change its lattice constant, as known for compliant layers. 

The reciprocal space map displayed in Figure 1f also shows a slight offset in the ‘Qx-axis’ direction between the peaks of the In_x_Ga_1−x_N and In_y_Ga_1−y_N layers, indicating that the In_y_Ga_1−y_N layer was about 20% relaxed compared to the In_x_Ga_1−x_N layer, with an ‘a’ lattice constant of 3.214 Å. This may be attributed to the formation of additional V-defects, as their density increased from 4.1 × 10^8^ cm^−2^ to about 4.8 × 10^8^ cm^−2^ after In_y_Ga_1−y_N layer regrowth, resulting in additional relaxation of the In_y_Ga_1−y_N layer. The V-defects often originate at the GaN/InGaN hetero-interface and their diameter increases with increasing layer thickness [27,28]. 

**Figure 2:**
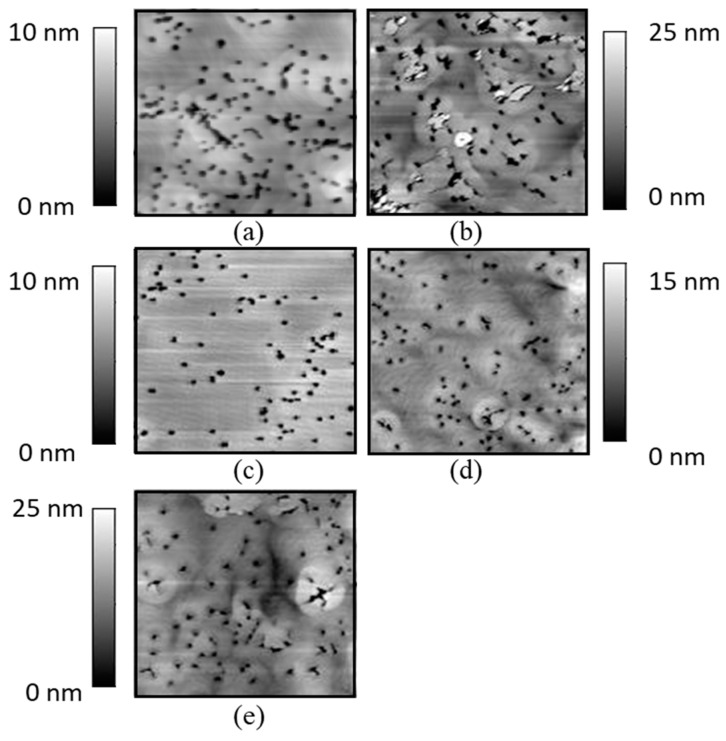
The 5 µm × 5 µm atomic force microscope (AFM) image of sample (**a**) A0 with 200 nm In_0.08_Ga_0.92_N before regrowth, (**b**) A1 upon regrowth of 100 nm In_y_Ga_1−y_N, (**c**) C0 with 80 nm thick In_0.09_Ga_0.91_N before regrowth, (**d**) C1 upon regrowth of 100 nm In_y_Ga_1−y_N, (**e**) C2 upon regrowth of 200 nm In_y_Ga_1−y_N.

The AFM image displayed in Figure 2a shows that the V-defect density was already relatively high, and larger V defects were visible upon the deposition of the 200 nm thick strained In_x_Ga_1−x_N (x = 0.08) layer. Upon regrowth of the 100-nm-thick In_y_Ga_1−y_N (y = 0.145) layer (Figure 2b) the V-defects started to coalesce, and additional defect features were visible on the surface. Similar defects had been observed for thick InGaN layers previously [29]. In view of the deteriorating surface morphology after 100 nm In_y_Ga_1−y_N deposition, regrowth on 200-nm-thick In_x_Ga_1−x_N layers was not further pursued.

### 3.2. Experiment 2: In_x_Ga_1−x_N Layer Thickness 80 nm, with Varying ‘x’ (Sample Series B, C and D)

In the following experiments, the thickness of the In_x_Ga_1−x_N layer was reduced to 80 nm to reduce both the size and the density of the V-defects. Three samples with indium mole fractions of 0.05 (referred to as sample B), 0.09 (referred to as sample C), and 0.12 (referred to as sample D) were grown. After tile fabrication and porosification (samples B0, C0, and D0), 100 and 200 nm thick In_y_Ga_1−y_N layers were regrown. The samples with 100 nm thick In_y_Ga_1−y_N layers will be referred to as samples B1, C1, and D1, and those with 200 nm thick In_y_Ga_1−y_N, as samples B2, C2, and D2.

**Figure 3:**
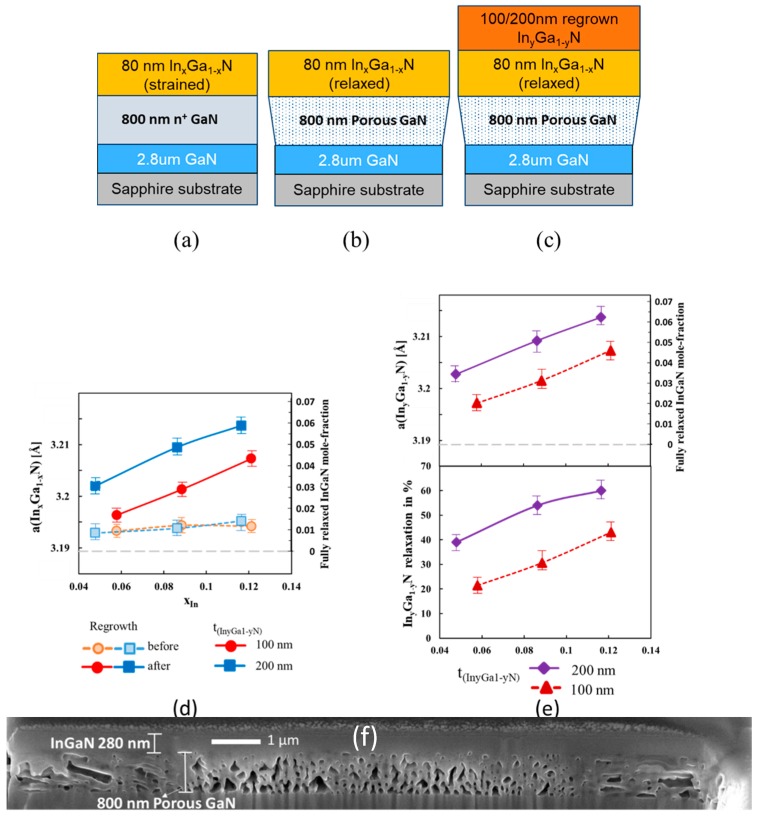
Epitaxial structure of samples with In_x_Ga_1−x_N layer thickness of 80 nm, with varying mole-fraction x (**a**) as-grown–samples B,C and D, (**b**) post porosification—sample B0,C0 and D0, (**c**) upon regrowth of 100 nm of In_y_Ga_1−y_N—samples B1, C1 and D1, and upon regrowth of 200 nm InGaN—samples B2,C2 and D2, (**d**) Lattice constant ‘a’ and corresponding In mole fraction of fully relaxed InGaN with the same lattice constant, after porosification, (open symbols, before regrowth, samples B0, C0 and D0), and after regrowth of 100 (filled circles, samples B1, C1, and D1) or 200 nm In_y_Ga_1−y_N (filled squares, samples B2, C2, and D2), versus mole fraction x in the as-grown In_x_Ga_1−x_N layers. Grey dashed line corresponds to the ‘a’ lattice constant of GaN, (**e**) (top plot) Lattice constant ‘*a*’ in angstrom, and corresponding mole fraction of a fully relaxed InGaN layer versus mole-fraction x of as-grown In_x_Ga_1−x_N layers, after regrowth of 100 (triangles) or 200 nm In_y_Ga_1−y_N layers (rhombi), (bottom plot). Degree of relaxation of In_y_Ga_1−y_N layers, versus mole-fraction of In_x_Ga_1−x_N layers, after regrowth of 100 (triangles) or 200 nm In_y_Ga_1−y_N layers (rhombi). Grey dashed line corresponds to the ‘a’ lattice constant of GaN, (**f**) Cross-sectional focused ion-beam SEM image of a 10 µm × 10 µm tile cleaved through the centre, for the sample C2 (200 nm In_y_Ga_1−y_N regrown on 80 nm of In_x_Ga_1−x_N, where x = 0.09 and y = 0.11).

The lattice constants obtained from the reciprocal space maps of samples B0, C0, and D0 taken after tile fabrication and porosification are displayed in Figure 3). The extracted lattice constants were 3.193 Å, 3.194 Å, and 3.194 Å for the In_0.05_Ga_0.95_N, In_0.09_Ga_0.91_N, and In_0.12_Ga_0.88_N samples, respectively. This lattice constant corresponds to a fully relaxed InGaN layer with mole-fraction 0.01–0.02. The small differences between the lattice constants reflected limited relaxation of the In_x_Ga_1−x_N layer for the three samples due to the thin In_x_Ga_1−x_N layer thickness. 

After regrowth of 100 nm In_y_Ga_1−y_N the samples were examined again (samples B1, C1, and D1). The measured indium compositions of the 100 nm thick regrown In_y_Ga_1−y_N layers were approximately 0.105, 0.11, and 0.12 for samples B1, C1, and D1, respectively. Note that these samples were co-loaded in the reactor to ensure the same growth conditions. The extracted ‘a’ lattice constants were 3.197 Å, 3.201 Å, and 3.207 Å, respectively (Figure 3e), corresponding to a degree of relaxation of the In_y_Ga_1−y_N layers of 21%, 31% and 43%, respectively (Figure 3e). 

After regrowth of the 100-nm-thick In_y_Ga_1−y_N layer, the lattice constants of the In_x_Ga_1−x_N layer increased as well, from 3.193 to 3.197 Å for samples B0/B1 (x = 0.05), from 3.194 to 3.201 Å for samples C0/C1 (x = 0.09), and 3.194 to 3.207 Å for samples D0/D1 (x = 0.12), as illustrated in Figure 3d. Comparing sample C1(lattice constant 3.201 Å) with sample A0 (lattice constant 3.202 Å), with similar mole-fraction and total InGaN layer thickness, we observe that sample C1 has nominally the same lattice constant but without the penalty of degraded morphology as can be observed from their AFM scans in Figure 2a,d, respectively.

For the samples with 200 nm thick In_y_Ga_1−y_N layers (B2, C2, and D2), which were all co-loaded in the reactor again, the following results were obtained (Figure 3d,e): the measured indium compositions of the 200-nm-thick regrown In_y_Ga_1−y_N layers were 0.106, 0.11, and 0.12 for samples B2, C2, and D2, respectively. The extracted ‘a’ lattice constants were 3.203 Å, 3.209 Å, and 3.214 Å, respectively (Figure 3e), corresponding to a degree of relaxation of the In_y_Ga_1−y_N layers of 39%, 54% and 60% (Figure 3e). 

Similar to the previous observations, upon regrowth of the In_y_Ga_1−y_N layer, the lattice constants of the In_x_Ga_1−x_N layer increased again, from 3.193 to 3.203 Å for samples B0/B2 (x = 0.05), from 3.194 to 3.209 Å for samples C0/C2 (x = 0.09), and 3.195 to 3.214 Å for samples D0/D2 (x = 0.12) (Figure 3d). The increase in In_y_Ga_1−y_N thickness resulted in additional stretching of In_x_Ga_1−x_N layer, which behaved as compliant layer due to its position on top of the porous GaN. In addition, the relaxation of the In_y_Ga_1−y_N layer itself increased, from 21% to 39% (samples B1/B2), 31% to 54% (samples C1/C2), and 43% to 60% (samples D1/D2) with higher thickness of the regrown In_y_Ga_1−y_N layer. With increasing In_y_Ga_1−y_N thickness, the nominal strain in the layer rose resulting in a stronger driving force towards relaxation. In elastic continuum theory, the strain energy per unit area, E_h_, for a pseudomorphic epilayer of thickness ‘h’ on a (0001) substrate, with misfit strain ‘ϵ’, shear modulus ‘G’ and poisson’s ratio ‘υ’ is given by [30]:

E_h_ = {2G × (1 + υ) ϵ^2^h} ÷ (1−υ). (2)

Considering the In_y_Ga_1−y_N layer as the epilayer and the In_x_Ga_1−x_N layer underneath as the substrate, the strain energy per unit area is directly proportional to the thickness of the In_y_Ga_1−y_N layer. When both layers are positioned on top of porous GaN, ‘a’ lattice constant of the In_x_Ga_1−x_N bottom layer is allowed to change and the strain energy can be lowered. For y ≥ x the ‘a’-lattice constant of the In_x_Ga_1−x_N layer increased leading to a reduction in the lattice mismatch between the In_y_Ga_1−y_N and In_x_Ga_1−x_N layers and a decrease of the misfit strain in Equation (2). This effect was more pronounced with the thicker In_y_Ga_1−y_N layer as the strain energy increased with thickness. To compensate for this strain energy increase, the degree of relaxation of the In_y_Ga_1−y_N layers increased (Figure 3e).

In addition, the decrease in lattice mismatch between the In_y_Ga_1−y_N and In_x_Ga_1−x_N layers with increasing x value resulted in an increase in the mole fraction y of the regrown In_y_Ga_1−y_N layers from 0.105 to 0.12 due to the composition pulling effect. Thereby the compositions measured for samples B1 and B2 with the lowest x value of 0.05 were similar to those obtained for the In_y_Ga_1−y_N layers on the corresponding GaN-on-sapphire reference samples, which amounted to y = 0.1 and y = 0.104 after 100 and 200 nm In_y_Ga_1−y_N regrowth, respectively.

### 3.3. Morphology Analysis Based on V Defect Density

While a higher indium mole fraction and thickness of the InGaN layers allowed the demonstration of layers with larger a lattice constants, the epitaxial parameter space was limited by the formation of defects. In MOCVD growth of strained In_x_Ga_1-x_N on GaN typically V-defects form in order to release strain energy [27,28,31]. Their density increases with increasing In_x_Ga_1−x_N composition and thickness (Equation (2)). As expected, the V-defect density on the surface of sample A with a 200- nm-thick In_0.08_Ga_0.92_N layer (Figure 2a), 4.1 × 10^8^ cm^−2^, was higher than that of 3.3 × 10^8^ cm^−2^ observed for sample C with 80-nm-thick In_0.09_GaN_0.91_layer (Figure 2c). As discussed earlier, when the V-defect density is high and the V-defects penetrate deep into the (In,Ga)N layer, strain relaxation can occur as the sidewalls of the V-defects are free to move to accommodate the strain, often resulting in a measurable enhancement of the In incorporation in the near surface region of InGaN layers (as observed for sample A1) or InGaN/GaN superlattices [32], due to the composition pulling effect. If the strain is too high, additional defects will form leading to a degradation of the InGaN layer properties, as observed for sample A1 (Figure 2b) in this study and as has been widely studied in the past [3,27,28,33,34].

In order to mitigate the degradation in layer properties when starting with 200 nm thick as-grown strained In_x_Ga_1−x_N layers (A series of samples), an alternate approach to achieve thicker InGaN layers was to start out with a thinner, only 80-nm-thick as-grown strained In_x_Ga_1−x_N layer with a lower V-defect density. Following with porosification of the GaN:Si underneath in order to allow partial relaxation of the In_x_Ga_1−x_N layer, and continuing the InGaN deposition process afterwards, utilizing the compliant property of the 80-nm-thick In_x_Ga_1−x_N layer during the deposition, resulted in a much lower V defect density. Sample C0 with 80-nm-thick In_0.09_Ga_0.91_N, for example, exhibited only 3.3 × 10^8^ cm^−2^ V-defects (Figure 2c) compared to 4.1 × 10^8^ cm^−2^ for sample A0 with 200-nm-thick In_0.09_Ga_0.91_N (Figure 2a). 

After depositing a further 100-nm-thick In_y_Ga_1−y_N layer with y = 0.11 on top of sample C0, the V-defect density on the surface increased only slightly to 3.5 × 10^8^ cm^−2^ (sample C1, Figure 2d) and was still lower than that of sample A0 (200 nm In_0.08_Ga_0.92_N, V-defect density 4.1 × 10^8^ cm^−2^). When increasing the In_y_Ga_1−y_N (y = 0.11) layer thickness to 200 nm (sample C2, Figure 2e), the V-defect density rose to 3.8 × 10^8^ cm^−2^, but was still lower than that of sample A0, despite its higher average indium composition of 0.105 and higher thickness of 280 nm, compared to sample A0 with an average indium composition of x = 0.08 and for the only 200 nm thick sample A0.

**Figure 4:**
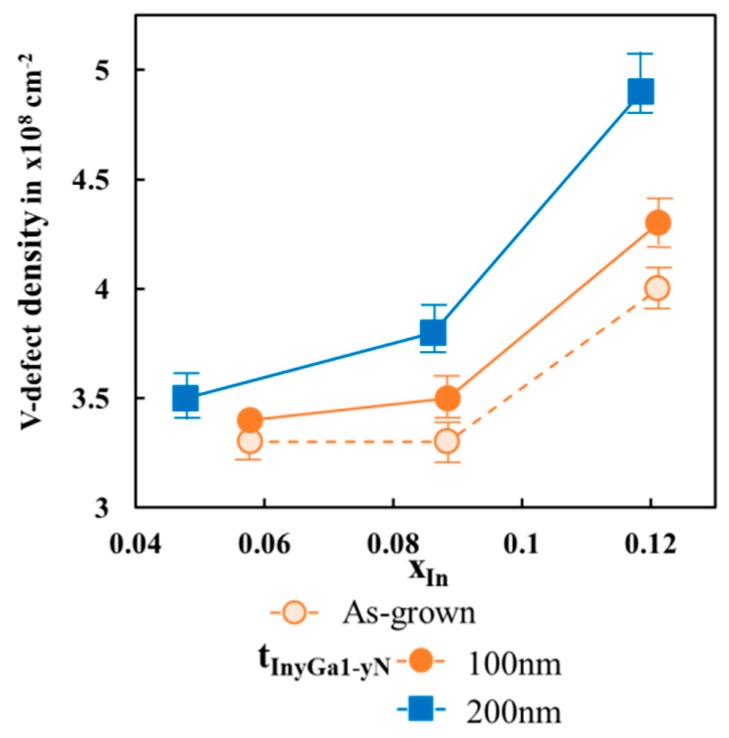
The average V-defect density of the sample surface vs. mole-fraction x of as-grown In_x_Ga_1−x_N layers, for as-grown samples (open circles), after regrowth of 100 nm In_y_Ga_1−y_N layers (filled circles) or 200 nm In_y_Ga_1−y_N layers (filled squares).

The average V-defect densities of all samples belonging to series B to D are shown in Figure 4. Note that upon porosification of the as-grown samples no change in the V -defect density was observed. For the as-grown samples with 80-nm-thick In_x_Ga_1−x_N layers, the V-defect density increased from 3.3 × 10^8^ cm^−2^ to 4 × 10^8^ cm^−2^ when increasing x from 0.05 to 0.12. Although the differences in v-defect densities may not seem drastic, it provides us with an overall guidance towards the trends to be observed for samples with different mole-fraction and total InGaN thickness.

As the In_x_Ga_1−x_N layers are initially grown coherently strained on top of the GaN:Si layers (samples B,C, and D) the strain energy increases with increasing mole fraction x, leading to a higher number of V-defects for MOCVD grown samples. Similarly, the number of V-defects in the regrown In_y_Ga_1−y_N layers increased with increasing x value, as the average composition of the combined layers, In_x_Ga_1−x_N and the In_y_Ga_1−y_N, rose as well. The more pronounced increase in V-defect density with increasing thickness of the In_y_Ga_1−y_N layer, samples B2, C2, and D2 versus samples B1, C1, and D1, can be attributed to the higher strain energy in the thicker layers. The circumstance, that once a V-defect has formed, it typically does not coalesce or fill under the growth conditions required for InGaN deposition, low temperatures, and the use of nitrogen as carrier gas in order to obtain sufficient indium incorporation in the MOCVD process, may contribute to this trend. For the above reasons, a low strain energy in the initially coherently strained In_x_Ga_1−x_N layers aided in achieving compliant partially relaxed In_x_Ga_1−x_N/In_y_Ga_1−y_N composite layers with low V-defect density. The degree of relaxation and the *a*-lattice constant of the In_x_Ga_1−x_N/In_y_Ga_1−y_N layer stack can be further increased through continued process optimization. In addition to thickness and composition of the layers on top of porous GaN, the degree of relaxation of the top layers is strongly dependent on the size of the etched patterns. Our previous study on stripe patterns showed that when the stripe width was reduced from 10 to 2 µm, the relaxation perpendicular to the stripes dramatically increased from about 60% to 100%, respectively [10]. The 10 µm × 10 µm tiles in this study were chosen for their compatibility with the micro-LED size regime. As micro-LEDs are pushed towards smaller sizes for applications in advanced micro displays, the pattern size for the InGaN pseudo-substrate fabrication process described here can be reduced as well, taking advantage of the larger lattice constants which can be obtained using smaller patterns. A detailed study towards the V-defect behavior and the relaxation mechanism of the InGaN layers on porous GaN is currently underway and will be presented elsewhere.

## 4. Conclusions

In conclusion, a method to fabricate compliant InGaN pseudo-substrates was demonstrated, allowing the fabrication of partially relaxed In_x_Ga_1−x_N/In_y_Ga_1−y_N (x = 0.09, y = 0.11) composite pseudo-substrates with an a-lattice constant of 3.209 Å, corresponding to fully relaxed InGaN with an indium mole fraction of 0.056. The presence of the porous GaN under-layer enabled the compliant behavior of the InGaN top layers. The fabricated InGaN pseudo-substrates are attractive for the fabrication of future electronic and optoelectronic devices.
